# Rotary Panoramic and Full-Depth-of-Field Imaging System for Pipeline Inspection

**DOI:** 10.3390/s25092860

**Published:** 2025-04-30

**Authors:** Qiang Xing, Xueqin Zhao, Kun Song, Jiawen Jiang, Xinhao Wang, Yuanyuan Huang, Haodong Wei

**Affiliations:** School of Mechanical Engineering, Nantong University, Nantong 226019, China; 2410310002@stmail.ntu.edu.cn (X.Z.); 2310320005@stmail.ntu.edu.cn (K.S.); 2210110004@stmail.ntu.cn (J.J.); 2315110245@stmail.ntu.edu.cn (X.W.); 2010110003@stmail.ntu.edu.cn (Y.H.); 17851072386@163.com (H.W.)

**Keywords:** irregular pipeline, pipeline imaging, full-depth-of-field imaging, large field of view, panoramic focusing image

## Abstract

To address the adaptability and insufficient imaging quality of conventional in-pipe imaging techniques for irregular pipelines or unstructured scenes, this study proposes a novel radial rotating full-depth-of-field focusing imaging system designed to adapt to the structural complexities of irregular pipelines, which can effectively acquire tiny details with a depth of 300–960 mm inside the pipeline. Firstly, a fast full-depth-of-field imaging method driven by depth features is proposed. Secondly, a full-depth rotating imaging apparatus is developed, incorporating a zoom camera, a miniature servo rotation mechanism, and a control system, enabling 360° multi-view angles and full-depth-of-field focusing imaging. Finally, full-depth-of-field focusing imaging experiments are carried out for pipelines with depth-varying characteristics. The results demonstrate that the imaging device can acquire depth data of the pipeline interior and rapidly obtain high-definition characterization sequence images of the inner pipeline wall. In the depth-of-field segmentation with multiple view angles, the clarity of the fused image is improved by 75.3% relative to a single frame, and the SNR and PSNR reach 6.9 dB and 26.3 dB, respectively. Compared to existing pipeline closed-circuit television (CCTV) and other in-pipeline imaging techniques, the developed rotating imaging system exhibits high integration, faster imaging capabilities, and adaptive capacity. This system provides an adaptive imaging solution for detecting defects on the inner surfaces of irregular pipelines, offering significant potential for practical applications in pipeline inspection and maintenance.

## 1. Introduction

Pipes are extensively utilized across a multitude of industries, including aerospace and fluid equipment. Owing to harsh working environments, their internal surfaces are highly susceptible to defects, including cracks, corrosion, fatigue damage, and coating delamination [1]. Diverse types of pipelines, with varying cross-sectional shapes, sizes, and other structural characteristics (e.g., aircraft intake pipes), present significant challenges in detecting internal pipeline anomalies. Consequently, developing an automated omnidirectional inspection device for detecting internal pipeline defects is crucial.

Existing pipeline defect detection technologies primarily encompass ultrasonic guided wave detection [2], magnetic flux leakage detection [3], Transient Test-Based Techniques (TTBT) [4,5], Spatial Transformation Based Multiscale Attention Network Detection (STMA-NET) [6], pipeline closed-circuit television (CCTV) detection [7], and machine vision-based detection.

Ultrasonic-guided wave detection technology uses the reflected signal generated by the guided waves at defects in the pipe wall for detection. The signal amplitude correlates with variations in the cross-sectional area of the pipe wall, enabling the determination of the pipe’s characteristics and condition through signal analysis and interpretation [8,9]. Nevertheless, this method faces significant challenges when applied to complex structures and diverse pipeline defects, as guided waves exhibit multi-modal characteristics and modal conversion effects, complicating signal transmission and analysis, and thereby hindering the precise identification of defect features [10]. Magnetic flux leakage detection involves magnetization of the pipeline so that the defects of the specimen form a leakage of the magnetic field, which is analyzed to identify anomalies [11,12]. However, this technology is limited to ferromagnetic pipeline materials and is unsuitable for surface-coated pipelines [13]. TTBT inspection detects internal leaks, pipe wall degradation, and other hidden problems through transient pressure wave analysis, which is suitable for undersea pipelines, long-distance water pipelines, and other environments where physical contact is difficult or poses a high risk. It can accurately locate the hidden internal position by using the reflection characteristics of the pressure wave [14]. The STMA-NET detection technology is mainly targeted at welding cracks, using X-ray images and deep learning models to improve the detection and generalization of complex cracks. However, these methods exhibit limitations in the comprehensiveness of weld defect detection, particularly in the quantification of tiny defects [15]. Traditional pipeline CCTV inspection technology systems, which require the placement of the camera equipment within the pipeline, display the internal image data as closed-circuit television recordings on the main controller. The inspectors analyze and determine the defects and conditions in real time. However, these methods have limitations, including difficulty in obtaining panoramic-image sequences owing to the limited camera view angle [16,17]. In addition, manual visual inspection is inefficient, costly, and prone to inaccuracies, and has a low degree of intelligence [18]. Furthermore, there are problems, such as difficulties in collecting data on pipeline defects and reproducing a complete view of pipeline interiors.

In contrast to the above techniques, pipeline imaging inspection technology offers distinct advantages, including clear results, accurate defect site measurements, and rapid detection capabilities. It is also generally applicable to all types of pipelines.

Machine vision-based pipeline imaging inspection technology is widely used [19], but conventional methods still face challenges when performing dynamic imaging of small-diameter or irregular pipelines [20]. Jing W et al. [21] used folded reflection panoramic imaging technology to extend the imaging perspective. This technique combines a camera with conical mirrors [22], hyperbolic mirrors [23], and other optical elements to overcome the limitations of a single view angle. The mirrors project a 360° panoramic image onto a two-dimensional plane [24]. However, in scenarios with complex variations in pipe diameters, the resulting unfolded panoramic image often suffers from severe distortion, and the correction algorithm fails, making accurate imaging difficult [25]. To enable effective panoramic imaging within diverse pipes with varying spans and adaptability, a radial 360° full-depth-of-field imaging device is needed. The device should offer a 360° field of view, both vertically and horizontally.

Additionally, most panoramic images of the inner wall of pipelines obtained with current methods display regular, symmetrical circular shapes [26,27]. However, the irregular cross-sectional shapes, imbalanced dimensions, and varying depths along the same axis in pipelines pose significant challenges [28,29]. Existing imaging devices struggle to meet the complex imaging requirements within pipelines.

This study proposes a rotary visual imaging device for panoramic depth-of-field imaging to address the challenges of imaging and inspecting the inner surfaces of pipelines. The device integrates scene depth detection and zoom imaging to address issues such as low imaging clarity, poor adaptability, and depth-of-field mismatches in existing devices, thereby improving the efficiency of panoramic imaging in irregular pipelines.

## 2. A Fast Circular Full-Depth Imaging Method Based on Depth Detection

**Figure 1:**
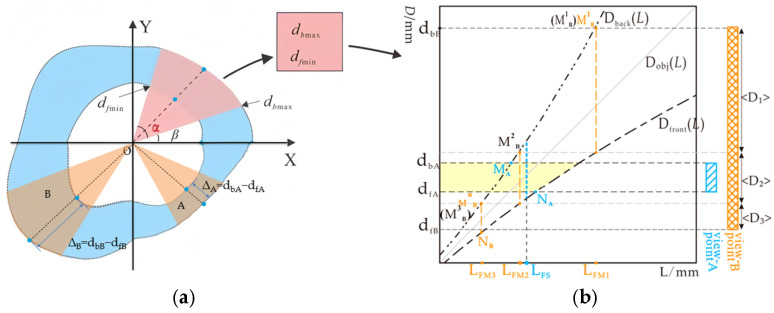
Full-depth-of-field imaging method based on depth distribution. (**a**) Schematic of annular panoramic large-angle imaging; (**b**) Basic principles of cross-mode full-depth-of-field imaging.

The radial cross-section of shaped pipes shows variations in radial span, and significant differences in depth of field occur across different view angles during imaging. Based on the camera’s depth of field and the focusing principle, achieving high-quality imaging of shaped pipe interiors requires aligning the pipe surfaces, which have varying depth spans, to the corresponding confocal surfaces within the depth interval. However, within the imaging field of view, the actual depths of both the nearest point and the farthest point in the depth span are substantial, making it impossible to image them on the same focusing plane. As a result, cameras with a single depth of field are often unsuitable [20]. Therefore, this study proposes a circular panoramic depth-of-field imaging method based on depth detection. The schematic representation of this principle is shown In Figure 1.

As shown in Figure 1, in the imaging process, firstly, the depth sensor acquires the circumferential 360° panoramic depth data, which are combined with the Gaussian imaging formulation of the depth-of-field model to generate the scene depth distribution curves (the foreground depth distribution curve d_f_ and the background depth distribution curve d_b_). Secondly, taking the view angle shown in Figure 1a as an example, based on the imaging sensor’s FoV (in horizontal view) angle α and the initial angle β, the circumferential scene depth distribution curve is divided into several sub-view angle intervals by the view angle segmentation algorithm.

The circumferential panorama is mapped as a polar coordinate system, and the 360° scene is segmented into eight sub-view angle intervals by taking the sensor optical center as the origin, using angle β as the starting angle, and angle α as the single view angle coverage angle. In each sub-view angle interval, the maximum value of background depth of field d_bmax_ and the minimum value of foreground depth of field d_fmin_ of the test view angle are extracted to calculate the depth of field span Δ = d_bmax_ − d_fmin_ of the interval.

Subsequently, the depth-of-field data of each sub-view angle are automatically analyzed using the cross-mode adaptive hybrid focus panoramic depth of field imaging method (Equation (1)) (Figure 1b):

1. Single-focus mode decision: If $D_{\mathrm{front}}^{-1}$(d_fmin_) ≥ $D_{\mathrm{back}}^{-1}$(d_bmax_) is satisfied, the sub-view can be categorized as a feature area (d_A_, d_B_), which only needs to be imaged by focusing once (number of imaging times M_i_ = 1).
2. Multi-focus mode decision: If $D_{\mathrm{front}}^{-1}$ (d_fmin_) < $D_{\mathrm{back}}^{-1}$(d_bmax_), then the target interval (d_fmin_, d_bmax_) should be divided into n sub-intervals by the depth segmentation algorithm (Equations (2) and (3)) (e.g., *n* = 3 corresponds to the intervals <D1>, <D2>, <D3>), and sequentially focus on the depth of the object plane D_FMj_ in each subinterval with the number of imaging times M_i_ = n.

(1) $$\{D_{F}\left(d_{f},d_{b}\right)\}=\left\{\begin{array}{l} \left\{D_{\mathrm{Fsingle}}(d_{f},d_{b})\},D_{\mathrm{front}}^{-1}(d_{f})\geq D_{\mathrm{back}}^{-1}(d_{b}\right)\\ \left\{D_{\mathrm{Fmulti}}(d_{F},d_{b})\},D_{\mathrm{front}}^{-1}(d_{f})<D_{\mathrm{back}}^{-1}(d_{b}\right) \end{array}\right.$$

(2) $$\left\{\begin{array}{c} \left(<D_{1}>\cup <D_{2}>\cup \ldots \cup <D_{n}>)\cap (d_{fB},d_{bB})=(d_{fB},d_{bB}\right)\\ n=N_{\min} \end{array}\right.$$

(3) $$D_{\mathrm{FMj}}=\left\{\begin{array}{c} D_{\mathrm{back}}^{-1}\left(d_{bB}\right),(j=1)\\ D_{\mathrm{back}}^{-1}(D_{\mathrm{front}}(D_{FM(j-1)})),(D_{f}(D_{FM(j-1)})>d_{fB}) \end{array}\right.$$

Following the above process, when the camera’s view angle (α) is fixed, varying the initial angle (β) generates different combinations of imaging positions. These combinations lead to various segmentation patterns, each with corresponding imaging modes and a total number of imaging operations (N = ∑M_i_) when capturing the circular panorama. Therefore, in the camera system’s automatic decision-making process, the choice of imaging modality is based on minimizing the number of focusing operations to achieve optimal imaging quality and efficiency.

## 3. System Design Overview

To improve the use of high-definition, full-depth-of-field imaging devices in complex and unstructured environments, a depth-rotating circular panoramic imaging device has been developed.

**Figure 2:**
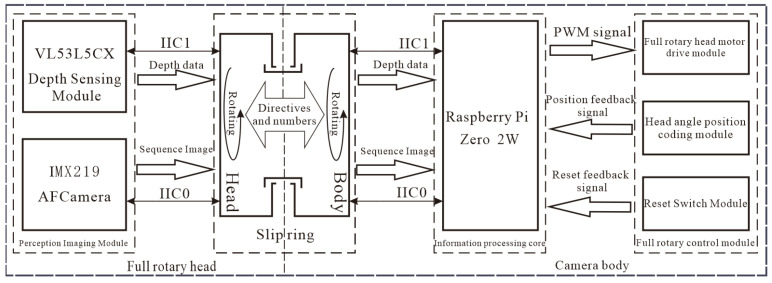
Block diagram of a panoramic deep rotational imaging system and data flow based on the Raspberry Pi Zero 2W.

This device is based on the Raspberry Pi Zero 2W (Raspberry Pi OS 64-bit) as the research and development platform. It features a fully automated hardware and software control system for image acquisition. The design integrates a depth perception module, an imaging module, an information processing module, and a rotation control module, as illustrated in the block diagram in Figure 2.

The device system consists of two main components: the full-rotation head and the camera body. The full-rotation head primarily includes a depth perception module and an imaging module. The rotary control module enables information acquisition in scenes with a large view angle. The camera body includes an information processing module and a corresponding rotary control and drive module.

## 4. Software and Hardware System Design

### 4.1. Overall System Hardware Design

**Figure 3:**
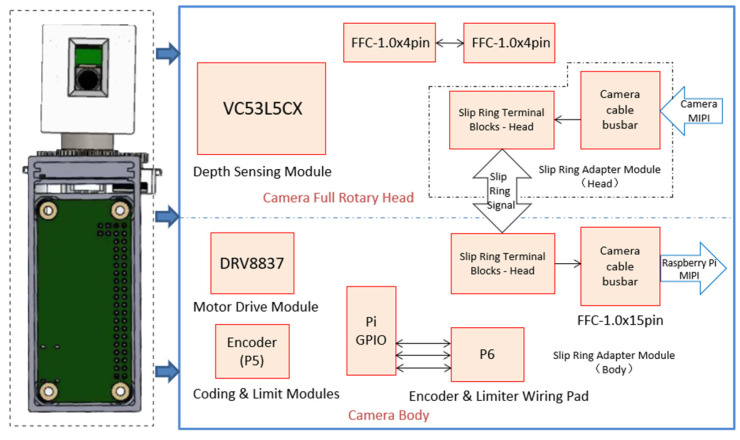
Schematic diagram of hardware circuit.

To achieve circular full-depth-of-field imaging, a 360° full-depth-of-field rotary vision imaging system with focus adaptation was independently developed, using a zoom camera with a specific view angle and a depth sensor. The principle of the hardware circuit is shown in Figure 3.

The system can be divided into three modules: depth-perception-based circumferential depth-of-field distribution acquisition, imaging, and information processing, according to its functional division. The depth-aware circumferential depth-of-field distribution acquisition module consists of two main components: a depth-aware module for detecting scene depth and a rotary control module, which includes a slip ring and motor to adjust the view angle of both the imaging and depth-aware modules under system control.

### 4.2. Depth-Perception-Based Circumferential Depth-of-Field Distribution Acquisition Module

The depth perception module utilizes a VL53L5CX ultra-miniature surface array depth sensor, capable of detecting distances up to 4000 mm within an effective range. It offers a diagonal field of view of 63° within the detection area, operates at a maximum frequency of 60 Hz, and features fully integrated, miniature characteristics, including multi-area, multi-object, and wide field of view capabilities.

The rotary control module is centered around a slip ring and utilizes motors, encoders, and limit switches to provide real-time feedback on the rotational angle position based on depth perception, forming a servo rotary control system. The slip ring allows continuous rotary motion of the camera and distance measuring modules while ensuring stable transmission of current and signals. This design promotes high system performance with a simple structure and prevents wires from twisting during rotation. The design of the differential signal lines and data transmission lines meets the requirements for transmitting data signals from the camera module, depth perception module, and Raspberry Pi Zero 2W. Channels DN0 and DP0, DN1 and DP1, and DN2 and DP2 form three sets of differential lines used for RGB control of the camera.

The incremental encoder provides real-time position feedback for full rotary drive and position control. It converts displacement into periodic electrical signals, which are then transformed into counting pulses, with the pulse count indicating the rotation angle. In this study, an EC12 encoder with 24 pulses and a resolution of 1.875° was employed. The encoder was driven by a slip ring via gear meshing, with a transmission ratio of 1:2. The position information was transmitted to the Raspberry Pi Zero 2W controller via pins A and B to enable closed-loop control and information feedback.

Limit switches were employed to calibrate the initial rotational position. They convert mechanical displacement into electrical signals and are essential for calibrating the camera’s initial zero position. The 3.3 V circuit channel and switch are connected to the microswitch, which remains at a high level in the normally open state. When the GND channel and switch are connected to the limit switch, it transitions to a low level in the normally closed state, marking the zero position for the distance measurement-camera module rotation. The generated signal was transmitted in real-time to the Raspberry Pi Zero 2W to obtain the initial rotational position information.

To address the rotational linkage between devices in this module, a hollow cup planetary gear motor was chosen, along with the DRV8837 driver chip. The DRV8837 is a highly integrated motor driver chip from TI, capable of regulating motor speed via the input PWM wave. Channels OUT1 and OUT2 are connected to the motor. When IN1 is high, and IN2 is low, the motor rotates in the forward direction; when IN1 is low, and IN2 is high, it rotates in the reverse direction.

**Figure 4:**
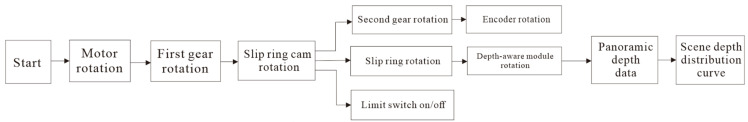
Workflow of acquisition module for circumferential scene depth distribution based on depth perception.

The workflow of the circumferential depth of the field-distribution acquisition module based on depth perception is shown in Figure 4.

When the motor starts, the first gear fixed on the motor output shaft drives the slip ring cam structure, and the second gear rotates through the transmission. This enables the depth perception module to rotate 360° along the slip ring’s rotational axis, measuring the distance of the circumferential pipeline, acquiring panoramic depth data, and generating the depth distribution curve of the scene within the pipeline. Using the sensor’s view angle α, the circumferential scene depth distribution curve is divided into panoramic view angles. The principle of depth segmentation is as follows: analyze and calculate the maximum depth (d_fmax_) and minimum foreground depth (d_bmin_) within the relevant segmentation view angle of the scene’s depth data and depth of field.

### 4.3. Imaging Module

The imaging module uses the SONY IMX219 (New York, NY, USA) zoom camera as the light-sensitive imaging element with a pixel size of 1.12 µm × 1.12 µm, a lens with a focal length of f = 4.0 mm, an aperture value of F = 2.0, and FoV = 54° × 41°. The focusing range is from 40 mm to infinity (∞), which is used for the calculation.

**Figure 5:**
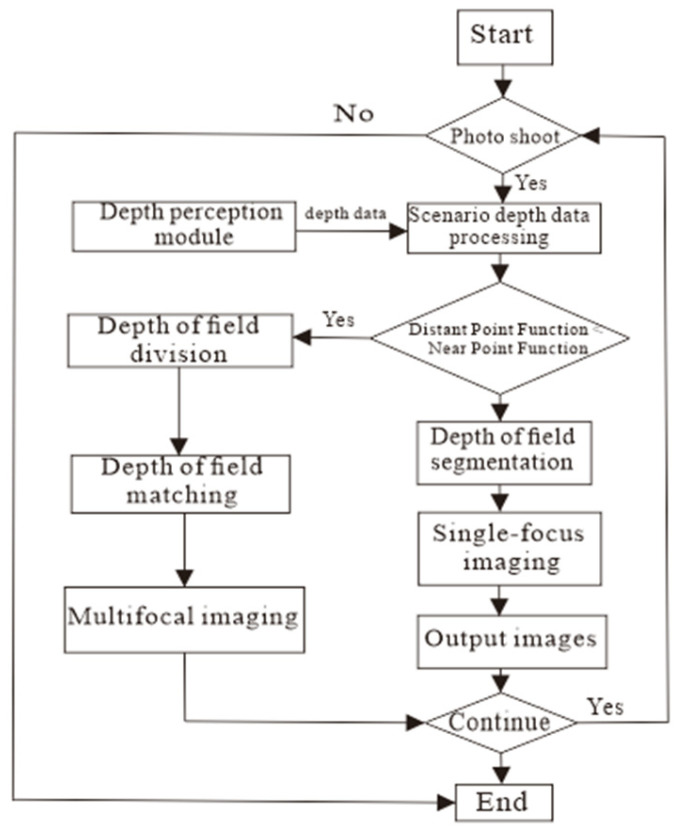
Diagram of single camera photographing control.

The module is based on the Raspbian Buster V10.0 operating system and performs panoramic sequence imaging at 45° intervals per view angle to capture panoramic mono-focus or multi-focus images. It utilizes the depth perception module to acquire processed depth distribution data. Based on this study, the system determines the focus mode for the current view angle. Within the corresponding mode, it matches the depth-of-field with the imaging depth interval, calculates the depth position of the confocal plane to obtain the required imaging, and performs either single-focus imaging or multi-focus imaging. The control flow for single-camera operation is shown in Figure 5.

### 4.4. Data Processing Module

**Figure 6:**
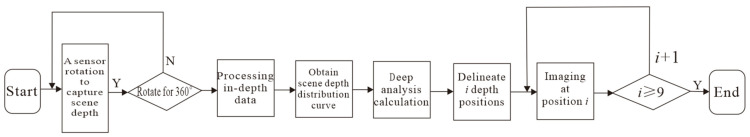
Diagram of the full depth of field imaging.

A full-depth-of-field rotary vision imaging system is needed to capture panoramic images of the pipeline’s inner surface. An automated acquisition system must be designed based on specific requirements to achieve data collection, transmission, image acquisition, splicing, and fusion. The information processing module uses a Raspberry Pi Zero 2W as the core processor and Python 3.9 for programming, which manages the depth perception, rotation control, and imaging modules. The collected depth data are processed by dividing the depth distribution region and combining the acquired panoramic image sequence, as shown in Figure 6.

First, the rotary control module, driven by the present module, operates the depth perception module, which acquires depth data of the pipeline’s inner surface through circumferential rotation. The acquired data are processed to divide the depth data into n ranges. Next, based on the depth data of the divided positions, the imaging module determines the hybrid focusing strategy for the corresponding depth range. The panoramic high-definition image sequence is captured and rotated using the rotary control module. Finally, the panoramic image sequence is spliced and fused to create a panoramic image of the pipeline’s inner surface.

## 5. Experimental and Analysis

### 5.1. Experimental Setup and Scene Configuration

**Figure 7:**
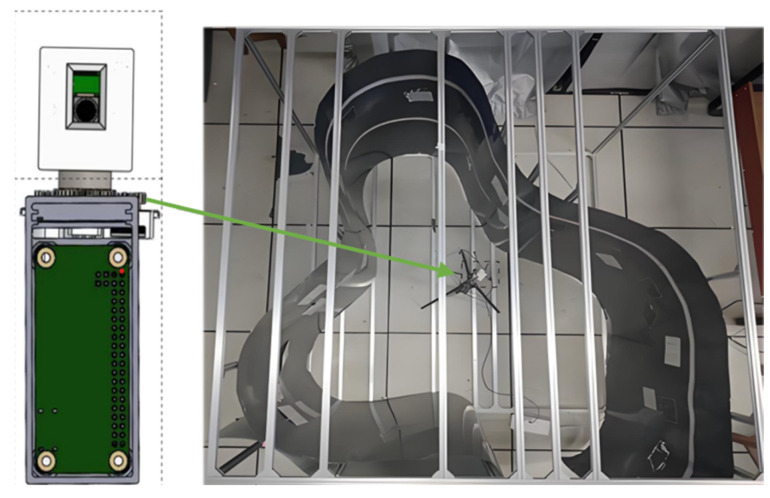
Rotary device of full depth of field imaging and experimental scene.

Figure 7 shows the 360° panoramic depth rotary imaging device and the scene inside the pipeline. To evaluate the panoramic imaging capabilities of the developed system, a shaped cavity pipeline model with a large span and continuous depth variation was constructed to simulate the inner surface of the pipeline with a wide depth of field. A free-form surface was constructed using thin grey iron sheets with a maximum depth of field of 960 mm. Additionally, “Appearance Inspection Grade Film” cards with varying widths of fine-grained features were attached to the inner surface of the pipeline model to assess the system’s imaging quality.

The 360° full-depth-of-field rotary imaging device was placed inside the shaped cavity pipe model, with the center of the square frame as the placement reference point. The surface coordinate system was initialized at 225°, serving as the starting angle for rotation and imaging. The device captured depth data within the pipeline and acquired panoramic sequence images. The panoramic depth was divided into depth distribution areas ①~⑧, corresponding to the sequence images of the pipeline’s inner surface captured by the device.

### 5.2. Panoramic Depth Acquisition and Imaging Experiment

The range of depth data acquired by the sensor at each view angle (areas ①~⑧) and the actual depth range of the heterogeneous pipeline model are shown in Table 1. The comparison results demonstrate that the developed device effectively adapts to pipelines with a wide depth-of-field distribution. It can adaptively acquire accurate depth data and capture the panoramic depth information of the pipeline. As an example, the depth data obtained from view angle ④, along with the depth re-segmentation and depth-of-field matching result, are shown in Table 1 and Table 2. Based on the confocal plane depth results, two focusing imaging processes are performed at 356 and 503 mm.

**Figure 8:**
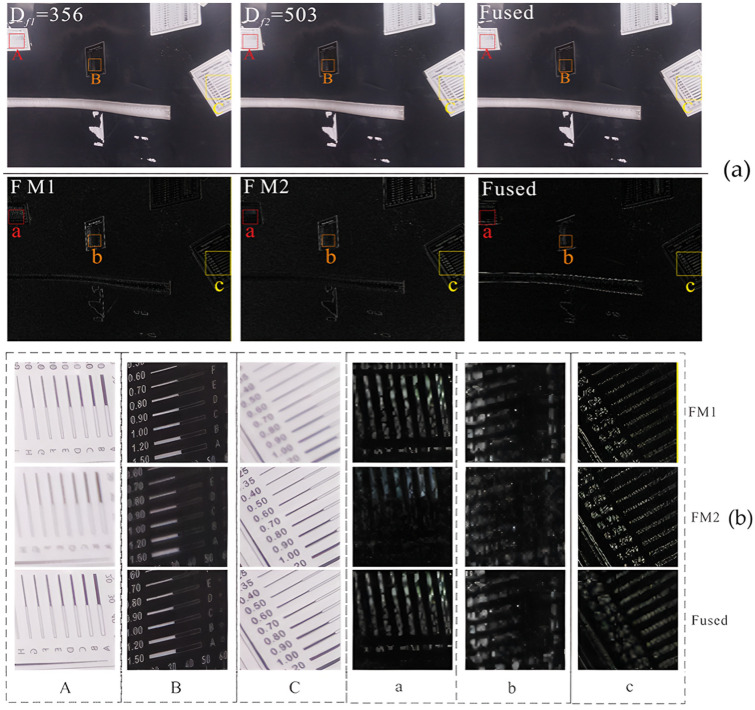
Comparison of fusion results of view angle ④. (**a**) Comparison of wavelet fusion results from view angle ④; (**b**) Comparison of texture details of view angle ④.

The multiple hyperfocus images acquired from a single view angle were aligned using the Fourier-Mellin algorithm [30], and multi-focus fusion experiments were performed using the fast wavelet fusion algorithm [31] to evaluate the quality of the fused images of the pipeline’s inner surface, utilizing enhanced high-frequency sub-band images. The full-focus fusion of view angle ④ is shown in Figure 8, accompanied by a texture detail comparison.

There are three regions, back region A, middle region B and front region C, which represent surfaces of different depths. D_f*i*_ (*i* = 1, 2) represents the images at two different confocal plane depths at view angle ④, and Fused is the fused image for D_f1_ = 356 mm and D_f2_ = 503 mm. The a, b and c are the images of the three regions of A, B and C after wavelet change. A comparison of the high-frequency features of the sub-band images before and after wavelet fusion shows that the high-frequency features in the fused image were distributed across all depth surfaces. In contrast, the high-frequency features of the pre-fusion image were limited to specific depth surfaces, with continuous and distinct differences. This demonstrates an improvement in the overall image clarity after fusion and the comprehensive recovery of the information on the pipeline’s inner surface.

### 5.3. Comparison and Analysis of Experimental Results

This study analyzes and evaluates the imaging performance of panoramic fusion-spliced images from both subjective and objective perspectives. The objective evaluation first relies on statistical image gradients and clarity assessments to calculate image sharpness.

The image gradient evaluation uses the gradient energy (EOG). The EOG function takes the square sum of the differences in gray value between a pixel and the adjacent pixels in the x- and y-directions as the gradient value of each, which is accumulated as input to the sharpness evaluation function. After averaging all pixels, the expression is:

(4) $$V_{EOG}=\frac{1}{X\times Y}\times \sum_{x}\sum_{y}\left\{{\left[g\left(x+1,y\right)-g\left(x,y\right)\right]}^{2}+{\left[g\left(x,y+1\right)-g\left(x,y\right)\right]}^{2}\right\}$$

The Vollath autocorrelation function reflects the similarity between two points in space. The edges of the clear texture details in an image are clear and sharp, and the correlation between the pixels is low, whereas the texture details in the out-of-focus area are blurred, and the correlation between image pixels is high. The calculation result reflects the similarity of all adjacent pixels, thus evaluating overall image quality. The Vollath function is expressed as follows:

(5) $$V_{\mathrm{Vollaths}}=\frac{1}{X\times Y}\times \sum_{x=1}^{X-2}\sum_{y=1}^{Y}\left[g\left(x,y\right)\times \left|g\left(x+1,y\right)-g\left(x+2,y\right)\right|\right]$$

In addition, the improvement in the detailed representation between the final image and each original image can be compared separately as a function of the signal-to-noise ratio (SNR) and PSNR [32]. SNR reflects the contrast between signal and noise in an image and is used to assess the noise suppression effect; PSNR represents the ratio of peak power to noise power in the fused image and can be used to measure the degree of distortion in the process of multifocal image fusion. The objective quantitative metrics were obtained using Equations (6) and (7).

(6) $$SNR=10\mathrm{lg}\frac{\sum_{x=0}^{X-1}\sum_{y=1}^{Y-1}p{\left(x,y\right)}^{2}}{\sum_{x=0}^{X-1}\sum_{y=1}^{Y-1}{\left[p\left(x,y\right)-q\left(x,y\right)\right]}^{2}}$$

(7) $$PSNR=10\mathrm{lg}\left[\frac{255^{2}}{\frac{1}{X\times Y}\sum_{x=0}^{X-1}\sum_{y=0}^{Y-1}{\left\|p\left(x,y\right)-q\left(x,y\right)\right\|}^{2}}\right]$$

In the evaluation functions of the Sections above, g(x, y) is the pixel value at (x, y) of the image in ①–⑧, p(x, y) is the pixel value at (x, y) of the original image, q(x, y) is the pixel value at (x, y) of the fused image, and X and Y are the numbers of rows and columns in the image pixel matrix, respectively. The calculation results are listed in Table 3 and Table 4.

The results of the objective assessment indicate that the clarity of images obtained through multi-focus sequence fusion is significantly improved compared to individual images before fusion. This is evident from objective quantitative metrics, such as clarity quantification, which demonstrate the advantage of the proposed imaging method in creating a panoramic representation of the pipeline’s inner surface across its full depth.

In addition, due to the difference in the depth of field of different view angles (e.g., the depth range of view angle ③ is 300–798 mm, and the depth range of view angle ⑧ is 725–872 mm), the number of focusing times and the difficulty of fusion are different, which in turn affects the effect of noise suppression.

**Figure 9:**
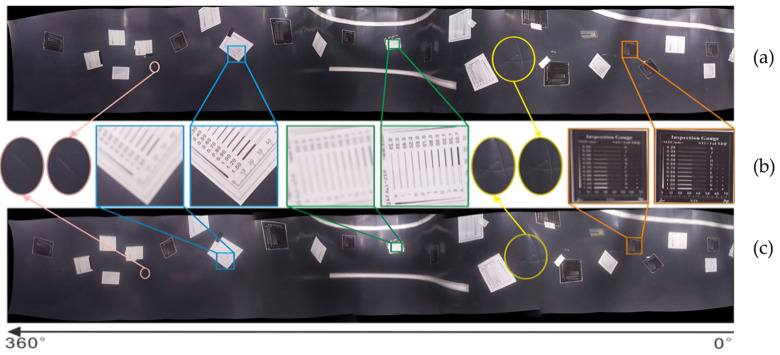
Panoramic view of the inner surface of pipeline model and subjective evaluation comparison. (**a**) Panorama stitching; (**b**) Local details and subjective comparative evaluation; (**c**) Panorama stitching of fixed-focus camera.

From a subjective angle of view, Figure 9a shows a panoramic image obtained by stitching and fusing images captured from eight view angles using the proposed imaging system. Figure 9c illustrates panoramic stitching of images captured from the same eight view angles using a fixed-focus camera. A zoomed-in comparison of the texture details of the two images is shown in Figure 9b.

By observing and comparing the image texture details at each depth distribution view angle of the two panoramic images, it is evident that the panoramic fusion map from a fixed-focus camera lacks sufficient clarity to display the full information of the pipeline’s inner surface, with fine lines on the inner wall being indistinguishable. In contrast, the panoramic depth imaging device developed in this study effectively captures the complete information of the pipeline’s inner wall and represents the global characteristics of the inner surface.

## 6. Conclusions

In this study, to address the limitations of existing conventional in-pipe imaging technology for heterogeneous or variable pipe diameters (e.g., aircraft intake pipes), a radial rotating panoramic imaging device with pipe size adaptability was designed alongside a fully automated hardware and software control system for image acquisition. The new panoramic imaging device comprises four modules: depth perception, rotation control, imaging, and information processing. Using a depth segmentation algorithm and full-focus imaging method, along with a micro servo rotating mechanism and circuit system, the device achieves circumferential 360° multi-view angle full-focus imaging, producing panoramic sequential images with complete focus of the pipeline’s inner surface. To verify the imaging effectiveness of the developed system, an experimental scene with a shaped free-form surface was constructed for panoramic depth imaging. The results demonstrated that the system quickly detects depth data of the pipeline’s inner surface and produces a high-definition, full-depth-of-field image containing complete inner wall information. This system has features such as high integration, fast imaging, and self-adaptation. It overcomes the limitations of existing internal pipeline surface imaging devices, such as complex structures, large size, and high costs, and can be widely applied to various types of complex curved surface scenes. Subsequent research will focus on further reducing the size of the device, enhancing the high temperature and corrosion resistance to make it adaptable to harsh environments such as petroleum and chemical industries, and at the same time optimizing the depth segmentation and image fusion algorithms to shorten the processing time to milliseconds.

## Figures and Tables

**Table 1:** Depth data range and imaging times for single view angle.

Statistical Indicators	Depth Ranges/mm	Number of Images/mm
①	725–960	1
②	629–960	1
③	300–798	3
④	357–634	2
⑤	320–797	3
⑥	338–797	2
⑦	338–872	3
⑧	725–872	1

**Table 2:** View angle ④ division of depth data distribution area.

Confocal Plane Number	Depth Segmentation Range	Depth of Confocal Plane
*i* = 1	311–417	356
*i* = 2	417–634	503

**Table 3:** Objective evaluation results of image clarity metrics (EOG and Vollath) of the images.

View Angle	EOG			Vollaths		
	V_EOG-FF_	V_Vollaths-AF_	SR	V_EOG-FF_	V_Vollaths-AF_	SR
1	106.2	193.3	82.0%	835.1	1033.4	23.8%
2	106.5	193.8	82.0%	745.0	842.6	13.1%
3	98.5	212.8	115.9%	694.4	870.5	25.4%
4	95.3	166.9	75.3%	669.9	830.4	24.0%
5	97.7	184.8	89.2%	689.7	855.6	24.0%
6	97.2	167.4	72.2%	729.0	948.8	30.1%
7	97.5	163.6	68.0%	587.9	750.9	27.7%
8	96.5	155.9	61.2%	742.2	852.4	14.9%

**Table 4:** Objective evaluation results of noise metrics (SNR and PSNR) of the images.

View Angle	SNR	PSNR
1	6.4	25.7
2	4.8	24.1
3	6.8	26.0
4	6.9	26.3
5	5.9	25.2
6	5.6	24.9
7	7.9	27.3
8	5.5	24.8

## Data Availability

All data and code will be made available on request to the correspondent author’s email with appropriate justification.
